# Prevalence of ear disease in small-breed dogs undergoing cone-beam computed tomography for dental procedures

**DOI:** 10.3389/fvets.2026.1822570

**Published:** 2026-04-22

**Authors:** Mahdi Haghighat, Georgia Mackay Burden, Hannah F. Boothe, Mary Krakowski Volker, Jason P. Hutt, Jennifer Tjepkema, Adrien-Maxence Hespel

**Affiliations:** 1 Animal Dental Center, Annapolis, MD, United States; 2 Animal Dental Center, York, PA, United States; 3 Animal Dental Center, Towson, MD, United States; 4 Animal Dental Center, Columbia, MD, United States; 5 Veterinary Dermatology Specialists, Perth, WA, Australia; 6 Veterinary Emergency Group, White Plains, NY, United States

**Keywords:** canine skull, cone beam computed tomography, dentistry, dog, ear disease

## Abstract

**Introduction:**

Following periodontal disease, ear disease is one of the most frequently reported diseases in dogs. Ear disease often remains undiagnosed due to the vague or absent nature of its clinical signs. Cone-beam computed tomography (CBCT), now widely used in the specialty of veterinary dentistry, enables concurrent assessment of ear structures during comprehensive oral health assessment and treatment (COHAT). This study evaluated the prevalence of ear disease in small-breed dogs undergoing CBCT during COHAT procedures.

**Methods:**

Dogs over 1 year of age and under 25.5 lbs. (~11.5 kg) that underwent CBCT as part of COHAT were prospectively enrolled and cross-sectionally evaluated. Imaging was reviewed for evidence of external, middle, and inner ear disease. Periodontal disease and tooth resorption were recorded to assess for potential associations.

**Results:**

Ear disease was diagnosed in 151/352 dogs (42.6%), predominantly involving the external ear canal (148 dogs). Thirty dogs had middle ear involvement, and three dogs had inner ear disease; some dogs had ear disease in multiple otic compartments. These findings exceeded previously reported clinical prevalence estimates of ear disease in dogs. No significant association was identified between CBCT-diagnosed ear disease and either periodontal disease or tooth resorption.

**Discussion:**

CBCT identified ear disease in nearly half of the evaluated dogs. Although no meaningful association was found between ear disease and dental pathology, these findings highlight the diagnostic value of CBCT beyond oral health evaluation in dogs.

## 1 Introduction

Following periodontal disease, ear disease is the second most commonly reported disease in dogs (1). The anatomical complexity of the canine ear canal and its surrounding structures, combined with the need for specialized equipment and diagnostic and therapeutic training, poses a challenge for accurate diagnosis of ear disease in small animals (2, 3). Recently, the introduction of high-resolution three-dimensional imaging, such as cone-beam computed tomography (CBCT) in veterinary medicine, has provided an opportunity to improve upon these diagnostic limitations. Recent studies using advanced imaging have demonstrated a high prevalence of ear disease and highlighted the significance of this modality for identifying ear pathology in dogs and cats (4–6).

Diagnostic imaging emerged in the field of human dentistry with traditional radiographs, followed by digital radiographs, implemented in 1896 and 1987, respectively (7, 8). The field further advanced when CBCT was introduced and became commercially available in the early 2000s (9, 10). Since then, CBCT has been progressively utilized in dentistry for the diagnosis of dental pathologies and for planning maxillofacial surgeries. It is used to create three-dimensional imaging and multiplanar reconstruction with high spatial resolution. In addition, quick acquisition time paired with a lower radiation dose for the patient, when compared to conventional multidetector computed tomography (MDCT), makes CBCT a preferred imaging choice in dentistry (11–13).

Recently, CBCT has become a valuable tool in veterinary dentistry, and several studies have compared this modality with conventional intraoral radiographs. In one study of small brachycephalic dogs, conventional radiographs showed inferior diagnostic yield in 9 out of 10 predefined diagnostic categories compared to CBCT (14). CBCT uses very small isotropic voxels that are suited for visualization of high-contrast mineralized structures, including teeth, maxillofacial bones, and the tympanic bullae. It offers lower soft-tissue contrast and is more susceptible to scatter and beam-hardening artifacts than MDCT or magnetic resonance imaging (MRI) (15–17). Furthermore, CBCT produces thinner, isotropic slices than MDCT, and has been described as providing higher spatial resolution for mineralized structures and improved delineation of fine dental anatomy (16, 17).

When CBCT is included as part of a Comprehensive Oral Health Assessment and Treatment (COHAT) procedure in a veterinary dental specialty center, capturing anatomical regions adjacent to the oral cavity, including the nasal cavity, sinuses, and ears, is inevitable. The field of view of the CBCT machine and the length of the dog’s skull will determine the extent of the additional anatomy surveyed. The inclusion of this anatomy should be acknowledged. This allows for the diagnosis of disease and promotes consideration as to how it may relate to dental pathologies. CBCT has been previously utilized in human otolaryngology for detailed assessment of temporal bone anatomy and for planning and evaluating implantation of bionic auditory devices. This demonstrates the diagnostic and clinical value of this modality for imaging the osseous structures of the ear (18). A few studies have also reported varying prevalences of ear disease in veterinary medicine using advanced three-dimensional imaging. In a recent prospective study of feline patients undergoing COHAT procedures, 41.4% were diagnosed with ear disease via CBCT (4). In another retrospective study of 177 brachycephalic dogs undergoing multidetector CT (MDCT) as part of the presurgical evaluation for brachycephalic airway syndrome, middle ear effusion was identified in 55 dogs (32%) (19). To our knowledge, no published studies have evaluated the prevalence of ear disease using CBCT in dogs. A previous study of dogs with lymphoplasmacytic rhinitis reported that approximately 55% of cases were suspected to have an underlying odontogenic origin (20) supporting the close anatomical and potential pathological relationship between dental structures and adjacent regions such as the nasal cavity and ear. Therefore, further investigation into potential associations between oral pathology and ear disease in dogs is warranted.

The primary objective of this prospectively enrolled cross-sectional study was to determine the prevalence of ear disease using CBCT in dogs that underwent a COHAT procedure at four veterinary dentistry specialty facilities. The secondary objective was to evaluate the potential association of ear disease diagnosed via CBCT with common dental pathologies, including periodontal disease and tooth resorption. The correlation of other variables, including age, body weight, and skull phenotype, with ear disease was also evaluated. We hypothesized that the large sample size of dogs in this prospectively enrolled cross-sectional study would provide a reliable estimate of the prevalence of ear disease and clarify the potential relevance of this condition to common dentoalveolar pathologies in dogs, using CBCT imaging.

## 2 Materials and methods

### 2.1 Study population and enrollment criteria

The initial population of this study consisted of 363 dogs over 1 year of age and weighing less than 25.5 lbs. (~11.5 kg) that underwent anesthesia for COHAT procedures at one of four veterinary specialty dentistry and oromaxillofacial surgery facilities (Towson, MD; Annapolis, MD; Columbia, MD; and York, PA) between June 15, 2024, and January 15, 2025. The age criterion was chosen because periodontal disease and tooth resorption are uncommon in dogs younger than 1 year of age, as these conditions typically develop following permanent tooth eruption and progress with age (21–23). The weight cutoff ensured full inclusion of both ears within the scan field of view (FOV), given the limitation (~13–14 cm) of the axial field of view of the CBCT units (VetCat Cone Beam CT) used in this study (24).

Based on the findings of Boothe et al. (4), who reported no significant influence of clinic location on the prevalence or distribution of ear disease, clinic site was not evaluated as a fixed-effect covariate in the present analysis. Cases from all four hospitals were pooled for primary analyses, and the clinic was modeled as a random intercept in the regression models to account for potential clustering at the practice level.

### 2.2 Exclusion criteria

Eleven dogs were excluded for the following reasons: five due to incomplete inclusion of the ears in the scan, two due to absence of rostral dentition, two for being edentulous, one due to inability to retrieve the file from the database, and one due to motion artifacts resulting in a non-diagnostic scan. This resulted in a final sample size of 352 dogs.

### 2.3 Owner consent and questionnaire

Clinical consent was obtained from the owners of all patients as part of the standard intake process. Consent forms were provided as either a hard copy or an electronic link at registration and included a signed release for imaging, photographs, and medical histories for research and publication purposes.

A brief questionnaire was originally used to collect information regarding any recent history suggestive of ear disease, including head shaking, ear rubbing, scratching, or ear discharge within 2 weeks before presentation. However, these data were excluded from statistical analyses because client-reported history has not been validated for diagnostic reliability, consistent with the methodology described by Boothe et al. (4). These questionnaire data were retained only for descriptive reporting and were not included in inferential analyses.

### 2.4 Imaging review

CBCT scans were interpreted using Invivo 7 software (25). Each file was evaluated by two AVDC diplomates (Dr. Hutt and Dr. Tjepkema) and a second–third-year veterinary dentistry resident (Dr. Haghighat) for the presence of tooth resorption and periodontal disease stages (PD2–PD4), based on AVDC classification (26). Periodontal disease stage 1 (gingivitis) was excluded, as it cannot be assessed by imaging alone. Tooth resorption and periodontal disease were further categorized by anatomic location as rostral (incisors and canines) or caudal (premolars and molars). Scans were randomly assigned among the three evaluators.

All scans were also independently reviewed by a veterinary intern (Dr. Burden) under the supervision of a board-certified radiologist (Dr. Hespel) for the presence or absence of ear disease, using the inclusion criteria originally defined by Boothe et al. (4): evidence of any mass lesion, canal stenosis, mineralization, hypoattenuating material consistent with fluid or soft tissue, osteolysis, or osteoproliferation affecting the external, middle, or inner ear canals.

**Figure 1:**
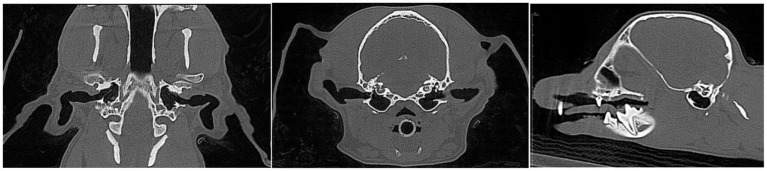
Multiplanar cone beam computed tomography (CBCT) images illustrate the normal anatomy of the tympanic bullae. **(A)** Dorsal, **(B)** transverse, and **(C)** sagittal reconstructions show symmetrical, well-aerated tympanic bullae with intact osseous margins and normal distinction between external, middle, and inner ear structures.

**Figure 2:**
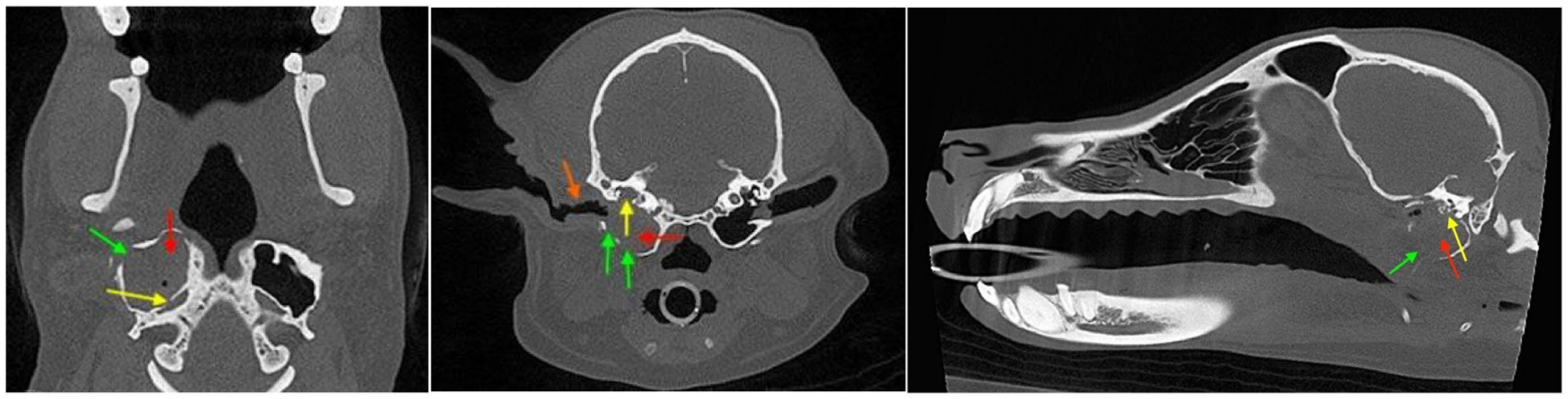
Example of middle and inner ear disease: multiplanar CBCT images of the right tympanic bulla showing osteolysis (green arrow), intraluminal soft tissue/fluid attenuation (red arrow), and irregularity of the promontory region (yellow arrow) consistent with inner ear involvement. On the transverse view **(B)**, there is also evidence of otitis externa in the right ear, with soft tissue thickening (orange arrow) compared to the contralateral side. **(A)** Dorsal, **(B)** transverse, and **(C)** sagittal multiplanar images illustrate loss of normal bulla wall definition and osseous change compared to the normally aerated contralateral side.

Figure 1 exemplifies the reference of a patient with normal ear anatomy in the current study, and Figure 2 illustrates middle and inner ear disease in a patient from this current study.

### 2.5 Variables

The correlation of demographic and morphometric variables, including age, sex, weight, and skull phenotype, with the presence of ear disease was evaluated at the univariable screening stage. Variables that did not meet criteria for confounding or statistical significance were not retained in the multivariable model.

The age variable was classified into three groups based on previously published age categories in domestic dogs that align with normative, age-linked physiological changes: 1–6 years (young/mature adult), 7–11 years (senior), and ≥12 years (geriatric) (27). The body weight variable was classified into three categories following growth standard references: <6.5 kg (toy breeds), 6.5–<9 kg (small breeds), and ≥9 kg (medium-sized breeds) (28), but did not remain in the final multivariable model.

The skull phenotype variable was classified based on the breed recorded in the medical record into four categories: brachycephalic (e.g., Pug, Boston Terrier, French Bulldog), dolichocephalic (e.g., Miniature Pinscher, Dachshund, Italian Greyhound, Welsh Terrier, Whippet), mesaticephalic (e.g., Beagle, Miniature Schnauzer, Welsh Corgi, West Highland White Terrier), consistent with previously published morphometric criteria (29, 30), and “undetermined” for mixed-breed dogs.

### 2.6 Statistical analysis

The primary outcome (presence of ear disease) was evaluated using an intercept-only generalized linear mixed-effects model (GLMM) with a binomial distribution and logit link. Associations between exposures of interest (rostral periodontal disease, caudal periodontal disease, rostral tooth resorption, and caudal tooth resorption) and ear disease were assessed using GLMMs with a logit link.

A univariable screening approach (*p* < 0.05) was applied to all exposure and confounder candidates (age category, weight category, sex, and skull phenotype). Variables that were statistically significant or that produced ≥30% change in the exposure effect estimate were retained in multivariable models. Two-way interactions between retained variables and the exposures of interest were evaluated. Owner-reported history of ear disease was excluded from inferential analyses due to its limited diagnostic reliability (4).

Age was retained as the sole confounder after screening because it demonstrated strong associations with dental pathology (periodontal disease and tooth resorption) and fulfilled epidemiologic criteria for confounding based on temporality and biological plausibility (21–23). Weight, sex, and skull phenotype did not meet confounding criteria and were therefore not included in the multivariable models. Age and weight were categorized based on previously published thresholds and nonlinearity observed via LOWESS plots against the logit of the outcome. Although weight was categorized and evaluated during screening, it did not remain in the final model.

All models included a random intercept for clinic location to account for potential clustering. A Tukey adjustment was applied to control for multiple comparisons. Analyses were conducted in R (version 4.3.3; R Core Team, 2024) (31) using the lme4 package (32) and the emmeans package (33).

## 3 Results

### 3.1 Prevalence of ear disease

The overall estimated prevalence of ear disease detected by CBCT in small-breed dogs undergoing dental procedures was 42.6% (151/352; 95% CI: 36.0–49.6%). External ear disease was identified in 148 dogs (99 bilateral, 49 unilateral), middle ear disease in 30 dogs (18 bilateral, 12 unilateral), and inner ear disease in 3 dogs (all unilateral). Some dogs had ear disease affecting multiple otic compartments, and therefore the sum of these categories exceeds the total number of affected dogs.

### 3.2 Univariable and multivariable analyses

In univariable analyses, only caudal tooth resorption (*p* = 0.01) was significantly associated with ear disease (Table 1). Neither rostral tooth resorption nor rostral and caudal periodontal disease were significantly associated with ear disease in the univariable analyses (Tables 2–4). Age was identified as a confounder of the association between caudal tooth resorption and ear disease. The interaction between age and caudal tooth resorption was not statistically significant (*p* = 0.07) (Table 5).

**Table 1:** Model-adjusted mean percent probability of ear disease by caudal tooth resorption 95% confidence intervals (CI), and a *p*-value of 0.01 from univariable models.

Caudal tooth resorption	
Exposure	Mean percent probability of an ear disease (95% CI)
Yes	50.1% (41.0–59.1%)
No	37.0% (29.5–45.2%)

**Table 2:** Model-adjusted mean percent probability of ear disease by rostral periodontal disease 95% confidence intervals (CI), and a *p*-value of 0.24 from univariable models.

Rostral periodontal disease score	
Exposure	Mean percent probability of an ear disease (95% CI)
0	27.9% (11.4–53.8%)
2	37.5% (23.8–53.5%)
3	50.5% (36.7–64.3%)
4	46.1% (35.1–57.4%)

**Table 3:** Model-adjusted mean percent probability of ear disease by caudal periodontal disease, 95% confidence intervals (CI), and a *p*-value of 0.12 from univariable models.

Caudal periodontal disease score	
Exposure	Mean percent probability of an ear disease (95% CI)
0	31.7% (15.0–54.8%)
2	33.0% (17.6–53.1%)
3	36.9% (21.6–55.5%)
4	49.0% (38.1–60.0%)

**Table 4:** Model-adjusted mean percent probability of ear disease by rostral tooth resorption, 95% confidence intervals (CI), and a *p*-value of 0.10 from univariable models.

Rostral tooth resorption	
Exposure	Mean percent probability of an ear disease (95% CI)
Yes	52.8% (39.0–66.1%)
No	42.4% (32.6–52.9%)

**Table 5:** Model-adjusted mean percent probability of ear disease by caudal tooth.

Caudal tooth resorption	
Exposure	Mean percent probability of an ear disease (95% CI)
Yes	48.1% (38.9–57.5%)
No	38.1% (30.4–46.4%)

Resorption after controlling for age. The *p*-value is 0.07 from multivariable models.

### 3.3 Other factors

The prevalence of rostral and caudal periodontal disease was 94.8 and 93.8%, respectively. Rostral tooth resorption was diagnosed in 23.6% of cases and caudal tooth resorption in 43.5%, with overall prevalence of periodontal disease of 97.4% and tooth resorption of 48.6%.

For the age variable, 104 dogs were 1–6 years, 138 dogs were 7–11 years, and 110 dogs were ≥12 years. The model-adjusted probability of ear disease increased with age: 33.8% (95% CI: 24.7–44.4%) in dogs 1–6 years, 43.9% (95% CI: 34.9–53.4%) in dogs 7–11 years, and 49.8% (95% CI: 39.6–60.0%) in dogs ≥12 years (*p* = 0.06), showing a trend of higher prevalence in older dogs.

The sex distribution was nearly equal, with 166 females and 186 males. The model-adjusted probability of an ear disease was 43.1% (95% CI: 34.6–52.0%) in females and 42.2% (95% CI: 34.2–50.8%) in males, with no significant association between sex and ear disease (*p* = 0.87).

The weight distribution included 199 dogs <6.5 kg, 94 dogs 6.5 to <9.0 kg, and 41 dogs ≥9.0 kg. The model-adjusted percent probability of an ear disease by weight category was 41.7% (95% CI: 33.8–49.9%) for dogs <6.5 kg, 43.4% (95% CI: 32.7–54.7%) for dogs 6.5 to <9.0 kg, and 44.7% (95% CI: 32.0–58.1%) for dogs ≥9.0 kg (*p* = 0.90), indicating no significant association between weight and ear disease.

Based on skull phenotype, 66 dogs were brachycephalic, 66 dolichocephalic, 98 mesocephalic, and 122 mixed/undetermined. The model-adjusted probability of an ear disease was 49.7% (95% CI: 36.9–62.6%) in brachycephalic dogs, 49.8% (95% CI: 36.9–62.7%) in dolichocephalic dogs, 43.8% (95% CI: 33.2–55.0%) in mesocephalic dogs, and 33.8% (95% CI: 24.9–44.0%) in mixed-breed dogs (*p* = 0.09), showing no significant association.

A total of 254 out of 352 owners (72.2%) responded to the questionnaire regarding recent ear disease. Fourteen owners (5.5%) reported a history of ear disease within 2 weeks prior to presentation, and 240 (94.5%) reported no history. Among the “Yes” responders, 8/14 (57.1%) had CBCT evidence of ear disease, while 6/14 (42.9%) did not. Among the “No” responders, 112/240 (46.7%) had ear disease, and 128/240 (53.3%) had none. For non-responders (unknown), 31.7% (95% CI: 23.0–42.0%) of dogs had ear disease.

Although descriptive data from the owner questionnaire were retained, this variable was excluded from statistical analyses due to the potential unreliability of self-reported otitis history, consistent with concerns raised in previous studies (4).

## 4 Discussion

This prospectively enrolled cross-sectional study showed a high prevalence of ear disease in small-breed dogs undergoing CBCT for dental procedures, with an overall prevalence of 42.6%. No significant correlation was identified between tooth resorption or periodontal disease and the presence of ear disease in the multivariable model analysis. This markedly high (42.6%) prevalence of ear disease in our cohort is substantially higher than previously reported clinical estimates of otitis externa, which have ranged from 7.3 to 20% (1, 34).

The prevalence of external and middle ear disease in our study was 41.6% (147/352) and 8.5% (30/352), respectively. The values reported in other studies using advanced imaging in dogs indicate a higher prevalence of external and middle ear disease. In one study that evaluated head and neck MDCT scans of dogs referred for reasons unrelated to ear disease, the prevalence of external and middle ear disease was reported to be 81.9 and 19.5%, respectively (6). In another study using MDCT in dogs with a history of chronic otitis externa, a high prevalence of middle ear disease (40.7%) was also reported (5). This discrepancy may be influenced by differences in study design, case selection, and imaging modalities.

While a direct causal relationship between caudal tooth resorption and ear disease is unlikely, it is important to consider why these conditions may occur concurrently in some dogs. In dogs, resorptive lesions develop when odontoclastic cells begin breaking down dental hard tissues, a process influenced by inflammation and age-related changes (23, 35). Middle ear disease also commonly reflects long-standing inflammation of the mucosal surfaces of the tympanic cavity and adjacent epithelial tissues, leading to mucosal thickening and impaired local defense and drainage (36, 37). Age is a recognized risk factor for otitis externa, with older dogs demonstrating higher rates and more severe grades of bacterial involvement (38). Similarly, the prevalence of tooth resorption increases with age (22, 23). Because both disorders become more common in older animals, their coexistence may reflect parallel age-related inflammatory processes rather than a direct causal relationship between them. The close physical relationship between the caudal maxillary teeth, the maxillary recess, and the middle ear, separated only by thin bone and interconnected vascular and lymphatic drainage, may further explain why inflammation in these regions may be detected concurrently (30). Considering these factors, the association observed in the univariable analysis is likely incidental.

Although odontogenic conditions (periodontal disease and tooth resorption) were not associated with ear disease in the final model, the possible biological effect of oral pathology on ear health via local inflammation or hematogenous spread cannot be ruled out. Periodontal disease is one of the most common progressive inflammatory conditions in dogs (21). This multifactorial condition involves a polymicrobial biofilm that progresses alongside the host’s immune response (39, 40). Several studies have identified organisms such as *Porphyromonas gulae*, *Treponema* spp., and *Fusobacterium* spp. within the canine periodontal microbiome, with variation in taxa depending on sampling and analytical methods (41–43). The association between periodontal inflammation and systemic inflammatory responses has been documented in human medicine, demonstrating that periodontal disease can have physiologic effects beyond the oral cavity (44–46). Reported associations between periodontal disease and altered inflammatory markers, renal biomarkers, and cardiovascular parameters further support the hypothesis that chronic oral inflammation may exert broader systemic effects (44–46). Metagenomic analyses of cerumen have demonstrated shifts in the ear microbiota of dogs with otitis externa toward opportunistic organisms such as *Staphylococcus* and *Pseudomonas* spp. (47). When considered alongside reports that anaerobic taxa such as *Fusobacterium* spp. can participate in infections involving both the oral cavity and upper aerodigestive tract (42, 48), these findings raise the possibility of partial microbial overlap between oral and otic ecosystems. Similarly, *Fusobacterium* spp. have been implicated in middle ear infections in human medicine (48), suggesting that certain anaerobic organisms may inhabit overlapping mucosal niches across oral and otic tissues. Despite this, in our study population, the high prevalence of odontogenic disease (periodontitis, 97.4%; tooth resorption, 48.6%) did not show any meaningful association with ear disease. The high incidence of dental pathology in our cohort likely reflects the referral-based nature of the study population.

Although CBCT is gaining popularity in veterinary dentistry, studies using this modality to estimate the prevalence of dental diseases in canines remain scarce. To date, there are no studies focusing only on the prevalence of tooth resorption or periodontal disease in canine patients. The prevalence of patients diagnosed with periodontal disease in our study was significantly high at 97.4%, with only nine patients from the total population showing no signs of periodontal disease on CBCT imaging. Due to the exclusion of stage 1 periodontal disease, which requires clinical evaluation, it is possible that all patients in this study had some degree of periodontal disease. This is consistent with previously reported prevalence of periodontal disease in canine patients, which has ranged between 44 and 100% depending on diagnostic methods and inclusion criteria (21, 49, 50). Based on the results of this study, the prevalence of tooth resorption was 48.6%, consistent with previously documented results from different studies evaluating this condition via radiographs. Peralta et al. found a prevalence of 53.6%, and Bae et al. documented 61.3% of dogs with resorptive lesions via full-mouth radiographs (22, 23). Variation in the reported prevalence of these odontogenic abnormalities across studies is likely due to differences in study populations, diagnostic criteria, and imaging protocols.

Due to the superior sensitivity and specificity of CBCT in the diagnosis of dental pathology, enhanced diagnostic detection has been documented in veterinary dentistry. In a study evaluating brachycephalic dogs, CBCT identified tooth resorption in 23.69% of evaluated teeth, showing higher detection rates than conventional intraoral radiographs (51). Similarly, in a study in human dentistry, the diagnosis of root resorption was reported in 15.7% of patients via CBCT, with many findings being incidental (52). This further highlights the importance of CBCT as a valuable diagnostic tool for detecting subtle lesions that may go unnoticed with oral radiography.

Weight and skull morphology are known risk factors in dogs with ear disease that have been previously investigated in several studies (53, 54). A retrospective study evaluating 868 dogs showed a lower prevalence of otitis externa in non-brachycephalic, intact female, and large-sized breeds (53). In addition, the alteration of the nasopharyngeal conformation of brachycephalic dogs has also been documented as a potential predisposing risk factor for auditory dysfunction and otitis media with effusion, particularly in Cavalier King Charles Spaniels (55, 56). These risk factors, particularly in our inclusion population, which is mostly composed of small and toy breed dogs with compact craniofacial anatomy, may affect drainage of otic fluid through the Eustachian tube, resulting in accumulation of sterile fluid in the tympanic bulla, a condition that might not be clinically apparent (56). However, our study did not demonstrate any significant link between these risk factors and the prevalence of ear disease diagnosed via CBCT. This could be due to the homogeneity of our sample pool, which primarily consisted of small-breed dogs referred for dental procedures, and the limited accuracy of skull-type classification based on the recorded breed in the medical record. In this study, 18.8% of dogs were brachycephalic, 18.8% dolichocephalic, 27.8% mesocephalic, and 34.6% mixed or undetermined. Future studies, including a more heterogeneous population and objective skull classification based on cephalic index measurement, may provide better insights into the association between these morphometric risk factors and ear disease.

In this study, owner-reported history of ear disease showed limited agreement with CBCT findings. Although descriptive analysis indicated that some dogs with reported otitis also had imaging evidence of ear disease, a large proportion of affected dogs were reported as clinically normal by their owners. This discrepancy may reflect the nonspecific or subtle nature of early ear disease in dogs and the limited reliability of self-reported questionnaires, as previously discussed by Boothe et al. (4). For these reasons, the questionnaire variable was excluded from the statistical analysis. Nevertheless, the descriptive data highlight the potential for under-recognition of ear disease by owners and reinforce the diagnostic value of CBCT in identifying clinically relevant pathology during dental procedures.

Several limitations should be acknowledged. First, the data collection period, which was only 6 months, precluded evaluation of potential seasonal influences on ear disease prevalence, in contrast to previous feline CBCT investigations that spanned a full year. Second, three evaluators (two diplomates and one resident) interpreted dental pathologies without formal inter-observer agreement testing, which may have affected diagnostic consistency. Nonetheless, the evaluators possessed advanced training in veterinary dentistry and oromaxillofacial surgery, strengthening the validity of the findings. A recently published study by Feigin et al. (57) highlighted variation in interpretation between residents and diplomates, underscoring the importance of standardized evaluation criteria. Third, the study population was derived exclusively from dentistry referral facilities rather than a random or general canine population, limiting the heterogeneity of the reported prevalence estimates. Fourth, while CBCT provided detailed visualization of external, middle, and inner ear structures, abnormalities were not corroborated or confirmed with cytology, culture, or myringotomy sampling; thus, imaging abnormalities cannot be equated with active clinical disease. Fifth, the inclusion of only dogs under 25.5 lbs. (~11.5 kg) was decided due to CBCT field-of-view constraints in our CBCT units, allowing complete visualization of both ears in a single scan, only in smaller patients. This may limit generalizability to larger-breed dogs. Finally, given the cross-sectional design of this study, no etiological or chronological link could be established between dental and ear diseases. These limitations emphasize the need for longitudinal, multi-institutional studies incorporating microbiological validation and a diverse sampling population to better define the clinical relevance of ear disease in canine patients.

A high frequency of ear disease was diagnosed by CBCT in small-breed dogs presenting for dental procedures, with an overall prevalence of 42.6%. These findings represent imaging abnormalities suggestive of ear disease and cannot be equated with active clinical disease. Despite the high prevalence of periodontal disease and tooth resorption in our study, these dental conditions were not independently associated with CBCT-identified ear disease. In the univariate analysis, only caudal tooth resorption was a significant variable. Collectively, these findings emphasize the diagnostic value of CBCT in identifying ear disease. To further clarify the clinical significance and potential pathophysiological connection between dental and ear disease in canine patients, a prospective, multi-institutional study integrating clinical assessment, microbiological correlation, and standardized imaging criteria is warranted.

## Data Availability

The raw data supporting the conclusions of this article will be made available by the authors, without undue reservation.
